# Ideals in the Operating-Theatre

**Published:** 1920-08-28

**Authors:** 


### Ideals in the Operating-Theatre.

The following passage from a war-time novel* proves that not all writers of fiction are blind to the ideals of doctors and nurses. It is a famous surgeon describing his methods during an operation: "The atmosphere in my theatre during an operation is like that of a church. We never speak a word—none of us. . . . We have discovered that anæsthetics are only partial in their operation—a very great discovery. The patient's normal consciousness is inhibited; but the other regions of consciousness are not. Handle an organ roughly and you see it wince. . . . So, you see, we men who cherish ideals and who love perfection as clergymen tell us they love God, work in absolute silence, and with the extremity of tenderness, and with all our thoughts solemnly directed, *solemnly*, to saving life. We are thinking of the whole patient, his consciousness, his unconsciousness, his soul. We are very, very tender; we are also reverent; perhaps some of us are praying. And you've no idea how great is our reward when we do an operation impossible ten years ago, and find our patient next day with no sign of shock. That's triumph. That's the wage God gives us."

* *An English Family*, by Harold Begbie. Page 198.

